# Disrupted joint action accounts for reduced likability of socially anxious individuals

**DOI:** 10.1016/j.jbtep.2019.101512

**Published:** 2020-09

**Authors:** Mia Maria Günak, David M. Clark, Miriam J.J. Lommen

**Affiliations:** a Department of Clinical Psychology and Experimental Psychopathology, University of Groningen, Netherlands; b Department of Experimental Psychology, University of Oxford, United Kingdom

**Keywords:** Social anxiety, Joint action, Social perception, Conversation

## Abstract

**Background and objectives:**

Motivated by their fear of disapproval, individuals with social anxiety continually monitor their own behaviour during social interactions hoping to prevent dreaded negative outcomes. Ironically, they do evoke less positive reactions from others. This study investigated whether lower engagement in the interpersonal process of joint action by socially anxious individuals leads them to attract less positive ratings by their conversation partners.

**Method:**

High socially anxious (HSA; *N* = 36) and low socially anxious individuals (LSA; *N* = 36) had separate conversations with a naïve conversation partner (*N* = 36). Conversations were filmed and analysed for joint action using the conventional manual way and a more exploratory automated way. Conversation partners rated the quality of the interaction and the person they talked to.

**Results:**

Conversation partners rated HSA participants less positively than LSA participants. The conventional manual method showed less joint action in conversations with LSA participants and crucially, joint action served as mediator between social anxiety status and general impression, quality of interaction and desire to meet again. These results were not replicated with the automated method.

**Limitations:**

The study used an analogue sample and future research should investigate whether these findings also apply to a clinical sample. Future studies should further explore the utility of automated techniques to measure joint action.

**Conclusion:**

Reduced joint action may explain why socially anxious individuals tend to be perceived less positively by others. The findings emphasise the importance of taking an interpersonal and holistic approach to understanding aspects of social anxiety disorder.

## Highlights

• Conversation partners perceive socially anxious participants less positively.
• Manually rated joint action is lower in socially anxious individuals.
• Joint action mediates relationship between social anxiety and positive ratings.
• Movement synchrony as measured by motion energy is associated with positive ratings.
• Movement synchrony as measured by motion energy is not related to social anxiety.

## 1 Introduction

Social anxiety disorder (SAD) at its core is the fear of scrutiny and negative evaluation by others (American Psychiatric Association, 2013; Clark & Wells, 1995). According to cognitive models of social anxiety disorder, people with SAD tend to hold dysfunctional beliefs about themselves and their social environment giving rise to a negative interpretation bias of social cues (Clark, 2005; Clark & Wells, 1995; Hofmann, 2007; Rapee & Heimberg, 1997). Another key factor that is implicated in the maintenance of SAD is focus of attention (Clark & Wells, 1995; Hofmann, 2007; Ingram, 1990; Rapee & Heimberg, 1997). During social interactions, socially anxious individuals show an increase in self-focused attention. They closely monitor how they think they are coming across to other people and often generate negative images or self-impressions of how they think they are seen by others (Clark, 2005; Clark & Wells, 1995; Rapee & Heimberg, 1997). At the same time, external attention is reduced (Spurr & Stopa, 2002) and the individuals are less able to detect positive social cues from others (Veljaca & Rapee, 1998).

Studies that have investigated the way in which socially anxious individuals are perceived by others during conversations have established two separate effects. First, socially anxious individuals tend to underestimate their performance compared to ratings made by their conversation partners (Hirsch, Meynen & Clark, 2004; Mansell & Clark, 1999; Rapee & Lim, 1992; Stopa & Clark, 1993). Second, there is some truth in their concern that they will be perceived less positively than non-anxious individuals. Interlocutors seem to find socially anxious individuals to be less likeable and comfortable (Meleshko & Alden, 1993), less friendly (Pilkonis, 1977), less warm and interested (Alden & Wallace, 1995), performing less well (Stopa & Clark, 1993), and interactions with them are less rewarding and pleasant (Heerey & Kring, 2007; Pilkonis, 1977). Consequently, conversation partners appear to have a reduced desire for future interactions (Papsdorf & Alden, 1998).

In the past decades, social skills deficits have been considered a likely explanation for the fact that socially anxious individuals tend to be rated less positively by others in conversations and other social interactions (Hopko, McNeil, Zvolensky, & Eifert, 2001; Trower, Yardley, Bryant, & Shaw, 1978). However, studies have failed to consistently establish that people with social anxiety disorder lack social skills (Mein, 2013; Segrin & Kinney, 1995). Instead, Clark and Wells (1995) have argued that self-focused attention and the use of safety-seeking behaviours are a more likely cause. If socially anxious individuals are excessively self-focused and pre-occupied with self-protective strategies, they may present themselves in a less favourable way and their internal pre-occupation may also interfere with the natural flow of an interaction.

One interpersonal process that might suffer from such depleted resources due to increased self-focused attention is the coordination between two individuals' actions, which serves as the basis of social connectedness (Marsh, Richardson, & Schmidt, 2009) and is important for establishing relationships (Lakens, 2010). Joint action is a process where two individuals predict and complement each other's behaviours to accomplish a common goal, promoting rapport (Clark, 1996; Pickering & Garrod, 2013; Sebanz, Bekkering, & Knoblich, 2006). Dialogue in language arises from a joint activity as well with the goal that what is said is not only listened to but also understood, moment-by-moment (Bavelas, Coates, & Johnson, 2000; Clark, 1996; Garrod & Pickering, 2009; Krauss & Weinheimer, 1967; Schober & Clark, 1989). Thus, a dialogue is a collaborative effort and not simply the sum of independent actions (Branigan, Pickering, McLean, & Cleland, 2007; Schober & Clark, 1989), out of which a temporarily coordinated social unity emerges (Lakens, 2010; Marsh et al., 2009; Marsh, Richardson, Baron, & Schmidt, 2006).

Mein, Faye and Page (2016) recently used a variant of Bavelas et al.’s (2000) “close call” conversation paradigm to investigate joint action in social anxiety. In a dyadic conversation paradigm, a narrator separately told an anecdote to high and low socially anxious listeners. Specific verbal responses from the listeners indicating that they were becoming involved in telling the story (supplying words, anticipating next steps, etc.) were coded as instances of joint action and were significantly less common when the listener was a high socially anxious individual. By contrast, generic responses indicating understanding on the part of the listener without co-production of the anecdote were not less common in high socially anxious individuals.

The present study is a further exploration of joint action in social anxiety. In contrast to Mein et al.’s (2016) focus on the coordination of verbal responses, we used two measures of movement synchrony to assess joint action within non-verbal responses. First of all, the conventional way of measuring joint action by manual behavioural analyses according to Bernieri (1988) was applied. Coordinated movement, or behaviour meshing, is the precise timing of actions to coordinate with the rhythm of the actions of another person (Bernieri, 1988; Mein, 2013) and is considered to be an index of the harmoniousness of interpersonal behaviours (Bernieri, Reznick, & Rosenthal, 1988). Second, a newer methodology using an automated measure (i.e., Motion Energy Analysis; Ramseyer & Tschacher, 2011) was used to see whether it also grasps the concept of joint action as defined. We also extended Mein et al.’s (2016) findings by using mediation analysis to formally assess whether reductions in joint action might explain why high socially anxious individuals are perceived less favourably by their conversation partners. Moreover, we intended to investigate whether there is an association between joint action and interactant's affectivity.

## 2 Method

### 2.1 Overview

High and low socially anxious individuals separately had a conversation with a third person (the conversation partner). Conversations were videotaped and conversation partners subsequently rated the conversation, the person they talked to, and their own feelings during the conversation. The order of conversations with low and high socially anxious participants was counter-balanced. Two different methods were applied to analyse the same visual recordings with regards to joint action: manual behavioural analyses by independent assessors and automated analyses using the Motion Energy Analysis (MEA) Software. Neither the conversation partner nor the independent assessors were informed about the level of social anxiety of the other person in the conversations.

### 2.2 Participants

Participants were recruited at the University of Oxford and in the general population using both online and offline advertisements (e.g., email circular, websites, posters). Exclusion criteria were a) age below 18 or above 65 years, b) severe levels of depression (a score of 29 or higher on the Beck Depression Inventory; BDI), c) suicidal ideation (a score of ≥2 on item 9 of the BDI); and d) currently receiving treatment for a psychiatric disorder or drug-related problem.

Using the Social Interaction Anxiety Scale (SIAS) as a screening instrument, forty volunteers who scored 19 or below on the SIAS (Mattick & Clarke, 1998) took part in the low socially anxious group (LSA), forty participants who scored 34 or above on the SIAS took part in the non-clinical, analogue high socially anxious group (HSA; Rodebaugh, Woods, Heimberg, Liebowitz, & Schneier, 2006), and forty volunteers who scored 22 or below on the SIAS took part as conversation partners (CP). The intention was to recruit individuals as conversation partner who were low socially anxious but slightly more to the middle on a continuous scale than those individuals who were on the two more extremes (i.e., LSA and HSA).

A pilot study was conducted with one triple (conversation partner, LSA and HSA participant), which hence was excluded from further analyses. Additionally, three triples were excluded as the third participant (i.e., LSA or HSA) in each did not show up on the day of testing, resulting in 36 triples (i.e., 36 recorded conversations) that were included in the analyses. The descriptives of the three groups are provided in Table 1. The LSA and HSA participants and the conversation partners were matched on sex and age as closely as possible. Apart from one triple, LSA and HSA always had the same sex. In 22 triples, they had a conversation with a CP of opposite sex and in 13 triples, they had a conversation with a CP of same sex. There were no group differences in sex, χ^2^(2, *N* = 108) = 4.44, *p* = .11, age, *F*(2,108) = 0.22, *p* = .80, first language, χ^2^(2, *N* = 108) = 2.37, *p* = .31, or level of education, χ^2^(12, *N* = 108) = 13.91, *p* = .31, where level of education was tested with maximum likelihood ratio Chi-square as assumptions of Pearson Chi-square were violated (McHugh, 2013).

**Table 1:** Characteristics of respondents.

	High socially anxious participants (*N* = 36)	Low socially anxious participants (*N* = 36)	Conversation partner (*N* = 36)
Sex (*female/male*)	19/17	20/16	27/9
*M(SD*) Age (*in years)*^1^	25.56 (6.82)	25.28 (7.69)	24.42 (8.03)
Level of education (*A-levels/Bachelors/Masters/Other*)	12/11/11/2	12/14/6/4	10/12/12/2
First language (*English/Other*)	21/15	27/9	25/11

^Note: 1^ missing value of two participants for age in the high socially anxious group.

### 2.3 Measures

#### 2.3.1 Beck Depression Inventory (BDI)

Beck Depression Inventory (BDI) is a well-established 21-items self-report measure of symptom severity of depression (Beck, Steer, & Brown, 1996). The inventory was used as a screening tool to ensure that participants were neither diagnosed with depression at clinically significant levels, nor suicidal.

#### 2.3.2 Social Interaction Anxiety Scale (SIAS)

Social Interaction Anxiety Scale (SIAS) was used as a screening tool to assess fear in interaction and conversation settings. The questionnaire consists of 20 items which are rated on a 5-point Likert scale ranging from 0 (*Not at all characteristic of me*) to 4 (*Extremely characteristic of me*; Alden & Mellings, 2004; Mattick & Clarke, 1998).

#### 2.3.3 Brief Fear of Negative Evaluation Scale, revised (BFNE-II)

Brief Fear of Negative Evaluation Scale, revised (BFNE-II) was used to assess the fear of negative evaluation (Carleton, Collimore, & Asmundson, 2007), which is a key feature of social anxiety disorder (Rapee & Heimberg, 1997). It comprises twelve items to be rated on a 5-point Likert scale ranging from 0 (*Not at all characteristic of me*) to 4 (*Extremely characteristic of me*; Carleton, McCreary, Norton, & Asmundson, 2006). The sum score of the BFNE-II was used for further analyses. In the present study, the internal consistency was excellent (α = 0.96).

#### 2.3.4 Liebowitz Social Anxiety Scale: Self-Report Version (LSAS-SR)

This 24-item scale was used to measure fear and avoidance of social interactional (11 items) and public performance (13 items) situations (Heimberg et al., 1999; Rytwinski et al., 2009). The participants were asked to rate fear and avoidance during the past week on two respective 4-point Likert subscales ranging from 0 (*None*) to 3 (*Severe*) for fear and from 0 (*Never, 0%*) to 3 (*Usually, 68–100%).* An overall score was obtained by summing the total scores of fear and avoidance. In the present study, internal consistency of the total scale was excellent (α = 0.96).

#### 2.3.5 Quality of Interaction Scale (QI)

Quality of Interaction Scale (QI) was used to assess how the participants evaluated the conversations they just had. For instance, to what extent participants thought that the interaction was smooth, strained, and pleasant (Berry & Hansen, 1996). The scale includes nine items and is rated on an 8-point Likert scale ranging from 1 (*Not at all*) to 8 (*Very much)*. The total sum score of the QI was used for further analyses. In the present study, only the QI filled out by the conversation partners were of relevance. The internal consistency was very good (α = 0.92).

#### 2.3.6 Desire for Future Interaction Scale (DFIS)

Desire for Future Interaction Scale (DFIS) measures the willingness to engage in future interactions with someone (e.g., whether one would like to meet that person, ask her/him for advice or sit next to her/him on a 3-h bus drive; Coyne, 1976). In the current study, this regarded the LSA/HSA person who the conversation partner just talked to. To reduce social desirability bias, the DFIS was modified in the study to a third-party perspective (Fisher, 1993; Mein, 2013). That is, the conversation partner was asked to imagine how the majority in the general population would respond to the partner based on first impressions. The DFIS consists of seven items to be rated on a 5-point scale ranging from 0 to 20% (*Very few people would)* to 81–100% (*Almost everyone would*). The total sum score of the DFIS was used for further analyses. In the present study, the internal consistency was good (α = 0.86).

#### 2.3.7 Global impression scale

Global impression scale (GI) was developed for the present study and includes three questions and can be rated on a visual analogue scale from 0 (*Very badly*) to 100 (*Very well*). To reduce social desirability bias on the part of the conversation partners, a third-party perspective was taken (i.e., “How would your partner come across to the average person?“, “What kind of impression would your partner make on the average person?“, “How likeable would the average person find your partner?“). The average score of the GI was used for further analyses. In the present study, internal consistency was excellent (α = 0.95).

#### 2.3.8 Positive and Negative Affect Scale (PANAS)

Positive and Negative Affect Scale (PANAS) comprises 10 items that assess positive affect and 10 items that assess negative affect. Each item is rated on a 5-point Likert scale from 1 (*Very slightly or not at all*) to 5 (*Extremely*; Watson, Clark, & Tellegen, 1988**)**. In the present study, internal consistency for both the positive and negative scales was good (α = 0.85 for positive scale and 0.75 for negative scale).

### 2.4 Procedure

After having provided informed consent, participants were screened online using the BDI and the SIAS. Those who met the inclusion criteria were then matched and invited to participate in the study. Participation was reimbursed with £7 or course credit for undergraduate students.

At the start of the experiment, participants completed additional measures of social anxiety: BFNE-II and LSAS-SR. Instructions for the study did not mention social anxiety. Instead, participants were told that the study investigated whether there is a relationship between a person's attention style and the kinds of thoughts and feelings they have about a situation. This cover story was used to increase the validity of the results as it was crucial that natural social interactions were observed.

Each test session involved two dyadic conversations for comparison between low and high anxious individuals having a conversation with a third person. Thus, a conversation partner (CP) had a 5-min conversation with the low socially anxious participant (LSA) or high socially anxious participant (HSA) separately, each in turn. Order of the conversations (HSA vs. LSA) was counterbalanced across triples. Conversation partners were told from the beginning that they will have two brief interactions with two individuals.

To start the first conversation, the CP and the first participant (i.e., either the HSA or LSA) were seated facing each other. The experimenter said that she needed to leave the room to set up further equipment in another room before beginning the experiment properly. The experimenter encouraged the participants to ‘talk among yourselves for a moment’ and assured them she will be back in 5 min to start the experiment, during which the participants actually were filmed already. This instruction was intended to help participants feel less conscious despite the video equipment, so that they behaved in the same way they would in an ordinary conversation outside of the lab. Cameras recorded whole body shots. After 5 min the experimenter came back into the room and told the participants that there was a problem with the equipment and that they would skip this part of the experiment but would try to solve it before the second conversation. Essentially the same procedure was repeated with the third participant (i.e., LSA or HSA in counter-balanced order). After each of the conversations, CPs rated their partner's social performance on the QI, DFIS and QI. These three measures together represented likeability with Cronbach's α = 0.74, indicating that they do overlap but also that there is no redundancy. Additionally, CPs completed a measure of their own positive and negative affect (i.e., PANAS). The testing session ended with the LSA and HSA participants watching the video footage of their conversations, while their eye-movements were recorded using an eye-tracking device. Afterwards, they rated their own social performance on the QI, DFIS, and GI. These last two parts of the testing session were unrelated to the topic of the current paper and will be reported elsewhere. Finally, participants were reimbursed and fully debriefed.

### 2.5 Behaviour analyses

Two independent assessors, blinded for the condition (i.e., LSA or HSA), viewed and coded the videos of the conversations. The first assessor rated all videos during the entire 5 min of each conversation. The second assessor rated a third of all videos, which were randomly selected. The videos were muted to facilitate rating movement and form.

#### 2.5.1 Joint action

The assessors used Bernieri's rating scheme (1988), which has been shown to be a valid method of measuring movement synchrony during social interactions (Bernieri, 1988; Bernieri, Davis, Rosenthal, & Knee, 1994; Bernieri, Reznik, & Rosenthal, 1988; Kimura & Daibo, 2006; Mein, 2013). On a 9-point Likert scale ranging from 1 (*Not at all*) to 9 (*Completely*), assessors were rated three items:

*Simultaneous movement*. Simultaneous movement reflects the degree and quantity of changes in movement that begin and stop, change speed or direction at the same exact moment. For instance, when the narrator starts to turn her or his head at the precise instant as the listener raises her or his face (Bernieri, 1988; Bernieri et al., 1988). Special attention is to be paid to the timing of the movements, where similarity of them is irrelevant.

*Tempo similarity*. Tempo similarity reflects the similarity of the interlocutors’ tempos of the actions. Assessors were asked to assume that all people have built-in tempos and rates of speed with which their actions and behaviour take place, just as an orchestra following a tempo at a concert. That is, the assessor rated the extent to which participants appear to march “to the beat of the same drummer” (Bernieri et al., 1988, p. 246).

*Gestalt-like smoothness of behaviour*. Gestalt-like smoothness of behaviour reflects a dance-like coordination regarding the degree of behaviour unity and the flow of the interactant's behaviours intertwining and meshing together, just as a choreographed dance.

For each video and assessor, a global joint action composite score was produced by taking the average (Bernieri, 1988; Bernieri et al., 1988; Kimura & Daibo, 2006) of the three above-mentioned scale scores. Internal consistency was good (α = 0.79). The composite score was intended to be used in all analyses.

### 2.6 Motion Energy Analysis

Motion energy analysis (MEA; Ramseyer & Tschacher, 2011; Version 4.03a) was used as a second method to quantify movement synchrony (i.e., joint action). The software allows to automatically and continuously monitor any changes of movement by detecting frame-by-frame changes in previously specified regions of interest (ROI) in the video footage of each conversation. For the present study, the CP's and their partner's (i.e., HSA or LSA) whole body, covering head and legs were defined as ROI, for a more detailed description see Supplementary Material.

## 3 Results

### 3.1 Social anxiety levels

As can be seen from Table 2, socially anxious individuals scored significantly higher than non-anxious individuals on the BFNE-II, *t*(69) = -7.54, *p* < .001, [-22.40, -13.02], *d* = 1.82, as well as on the LSAS-SR, *t*(69) = -8.11, *p* < .001, [-43.55, -26.36], *d* = 1.92. CP scored lower than HSA, *t*(69) = -6.17, *p* < .001, [-37.86, -19.61], *d* = 1.47, and similarly to the LSA on the LSAS-SR, *t*(70) = 1.47, *p* = .15, [-2.09, 14.54], *d* = 0.35. On the BFNE-II, CP scored slightly higher than the LSA, *t*(70) = 2.31, *p* < .05, [0.86, 10.47], *d* = 0.55, but lower than the HSA, *t*(69) = -4.68, *p* < .001, [-17.09, -7.00], *d* = 1.11.

**Table 2:** Means and standard deviations of scores of BNFE-II and LSAS-SR of HSA, LSA and CP.

	HSA (*N* = 35)^a^	LSA (*N* = 36)	CP (*N* = 36)
	*M* (*SD*)	*M* (*SD*)	*M* (*SD*)
BFNE-II^a^	31.69 (10.37)^a^	13.97(9.43)^b^	19.64 (11.28)^c^
LSAS-SR^a^	66.43 (19.77)^d^	31.47(16.42)^e^	37.70 (19.44)^e^

Note: HSA = high socially anxious; LSA = low socially anxious; CP = conversation partner; BFNE-II = Brief Fear of Negative Evaluation Scale, Revised; LSAS-SR = Liebowitz Social Anxiety Scale: Self-Report Version.

Non-matching numbers in a row indicate a significant difference, *p* < .05.

a Missing values of one participant for BNFE-II and LSAS-SR in the SA group.

### 3.2 Reliability of manual behaviour analyses of joint action

Interrater reliability between the two assessors for the three subscales of joint action, namely, simultaneous movement, tempo similarity and gestalt-like smoothness, were α = 0.62, 0.37, and 0.86, respectively. Given the low interrater reliability of tempo similarity, we decided to exclude the ratings of this subscore from further analyses. The global joint action score therefore from now onwards was the average of the score of simultaneous movement and gestalt-like smoothness. This resulted in an interrater reliability of the global joint action score of α = 72. which is comparable to what was found in earlier studies (Bernieri, 1988; Bernieri et al., 1994; Bernieri et al., 1988; Mein, 2013). Ratings of the global joint action composite score of the first assessor were used for further analyses. Internal consistency of simultaneous movement and gestalt-like smoothness scores was good (α = 0.81)

### 3.3 Conversation partner impressions

A multivariate analysis of variance (MANOVA) was used to investigate whether high social anxiety individuals are less liked or elicit less positive reactions from their conversation partners, as assessed by the DFIS, QI, GI and PANAS. As expected, there was a significant multivariate effect of social anxiety group (HSA vs. LSA), *F*(5, 66) = 2.77, *p* = .025; Wilk's Λ = 0.83, *Ƞ*_*p*_^*2*^ = 0.17. Univariate tests showed that CP rated HSA lower than the LSA on the quality of the interaction, their general impression of the person they were talking with, and their desire to meet that person again (see Table 3).^3^ Effect sizes for these findings are considered to be medium (Cohen, 1992). However, conversations with HSA and LSA did not differ significantly in the strength of either positive or negative emotions elicited in the CP (see Table 3). There was no evidence of an effect of same- or mixed-sex across groups on the conversation partner impressions in terms of the DFIS, *t*(70) = -1.59, *p* = .116, [-3.82, 0.43], for the QI *t*(70) = -1.95, *p* = .056, [-6.71, 0.08], and for the GI *t*(70) = -1.19, *p* = .239, [-11.59, 2.94], respectively.

**Table 3:** Partner impressions and joint action ratings of HSA and LSA.

	HSA (*N* = 36)	LSA (*N* = 36)				
	*M* (*SD*)	*M* (*S*D**)	*F*(1, 70)	95% *CI*	*p-*value	Cohen's *d*
DFIS	23.17 (4.29)	25.69 (4.25)	6.31	[0.52, 4.54]	.014	.59
QI	40.47 (7.88)	43.89 (5.92)	4.33	[0.14, 6.70]	.040	.49
GI	71.14 (16.72)	79.11 (12.04)	5.39	[1.12, 14.82]	.023	.55
Positive PANAS	28.81 (6.79)	29.28 (7.15)	.08	[-2.81, 3.75]	.775	.07
Negative PANAS	11.36 (2.10)	12.03 (2.81)	1.30	[-0.50, 1.83]	.258	.27
Joint Action	6.06 (1.51)	6.69 (1.05)	4.36	[0.03, 1.25]	.040	.49

Note: HSA = High socially anxious; LSA = low socially anxious; DFIS = Desire for Future Interaction Scale; QI = Quality of Interaction Scale; GI = General Impression Scale; PANAS = Positive and Negative Affect Scale.

### 3.4 Joint action

A one-way Analysis of Variance (ANOVA) was used to analyse the degree of joint action during each interaction. Joint action was lower in conversations with socially anxious participants than in conversations with non-anxious participants according to the manual behavioural analyses (see Table 3).

### 3.5 Mediation analyses

To test whether joint action mediates the relationship between social anxiety and the three facets of likeability, a mediation analysis was conducted for each of the conversation partner impression scales that had a significant relationship with social anxiety group (i.e., DFIS, QI, and GI). PANAS was associated neither with social anxiety (Table 3), nor joint action itself: positive items, *b* = 0.12, *t*(69) = 0.184, 95% CI [-1.13, 1.36], *p* = .855, and negative items, *b* = -.25, *t*(69) = -1.12, 95% CI [-0.69, 0.19], *p* = .265. Mediation analyses were conducted with PROCESS macro (Hayes, 2012).

#### 3.5.1 Mediation analysis of the desire for future Interaction Scale (DFIS)

**Fig. 1:**
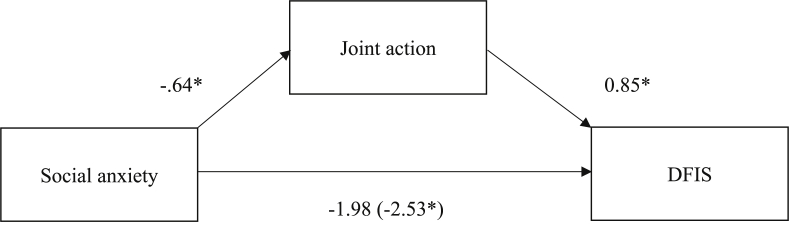
Joint action as a mediator between social anxiety and DFIS. Note: DFIS = Desire for Future Interaction Scale *p < .05.

The mediation analysis revealed that social anxiety was a significant predictor of the DFIS score, *b* = -2.53, *t*(70) = -2.51, 95% CI [-4.54, -0.52], *p* = .014, *R*^2^ = 0.08. Social anxiety was also a significant predictor of joint action, *b* = -.64, *t*(70) = -2.13, 95% CI [-1.25, -0.03], *p* = .041, *R*^2^ = 0.06. In turn, joint action, was a significant predictor of desire of future interaction, *b* = 0.85, *t*(69) = 2.23, 95% CI [0.09, 1.62], *p* = .03. When the indirect effect of social anxiety on DFIS via joint action was taken into account, the direct effect of social anxiety on DFIS was no longer significant, *b* = -1.98, *t*(69) =-1.96, 95% CI [-4.00, 0.03], *p* = .054. These results provided support for partial mediation (Fig. 1). Approximately 14% of the DFIS variance was accounted for by both predictors (*R*^2^ = 0.145). The bootstrap estimation approach with 5000 samples revealed that the indirect effect of joint action was significant, *b* = -.55, 95% CI [-1.45, -0.06]. Thus, being socially anxious was associated with 0.55 points lower scores on DFIS as mediated by joint action.

#### 3.5.2 Mediation analysis on for quality of interaction (QI) ratings

The mediation analysis with QI as dependent variable revealed similar results. Social anxiety predicted QI, *b* = -3.42, *t*(70) = -2.08, 95% CI [-6.69, -0.14], *p* = .041, *R*^2^ = 0.06, and joint action, *b* = -.64, *t*(70) = -2.09, 95% CI [-1.25, -0.03], *p* = .041, *R*^2^ = 0.06. Furthermore, joint action predicted QI, *b* = 1.70, *t*(69) = 2.77, 95% CI [0.48, 2.92], *p* = .007. More importantly, social anxiety no longer predicted QI when the indirect effect via joint action was taken into account, *b* = -2.33, *t*(69) =-1.44, 95% CI [-5.56, 0.89], *p* = .154. Therefore, joint action partially mediated the relationship between social anxiety and QI. Approximately 15% of the variance in QI was accounted for by both predictors (*R*^2^ = 0.153). The bootstrap estimation approach with 5000 samples revealed that the indirect effect of joint action was significant, *b* = -1.08, 95% CI [-3.28, -0.06]. This means that, being socially anxious was associated with 1.08 points lower scores on QI as mediated by joint action.

#### 3.5.3 Mediation analysis for on global impression (GI) ratings

The third mediation analysis revealed that social anxiety predicted GI, *b* = -7.97, *t*(70) = -2.32, 95% [-14.82, -1.12], *p* = .023, *R*^2^ = 0.07, and joint action, *b* = -.64, *t*(70) = -2.09, [-1.25, -0.03], *p* = .041, *R*^2^ = 0.06. Joint action also predicted GI, *b* = 2.97, *t*(69) = 2.28, [0.37, 5.57], *p* = .026. When the mediator (joint action) was taken into account, social anxiety no longer predicted GI, *b* = -6.08, *t*(69) = -1.77, [-12.94, 0.78], *p* = .081. Hence, joint action partially mediated the relationship between social anxiety and GI. Approximately 14% of the variance in GI was accounted for by both predictors (*R*^2^ = 0.136). The bootstrap estimation approach with 5000 samples revealed that the indirect effect of joint action was significant; *b* = -1.89, 95% CI [-6.37, -0.05]. Therefore, being socially anxious was associated with 1.89 points lower scores on GI as mediated by joint action.

### 3.6 Movement synchrony by MEA

Interestingly, no evidence was found for an association between social anxiety and movement synchrony as measured by frame-differencing, *F*(1, 70) = 0.07, 95% CI [-0.011, 0.008], *p* = .789. In fact, the mean difference of observed movement synchrony differs only about 0.001 between the high (*M* = 0.119, *SD* = 0.024) and low socially anxious participants (*M* = 0.120, *SD* = 0.017). However, movement synchrony appeared to have an effect on evoking positive reactions by their conversation partner. That is, movement synchrony was positively associated with DFIS, *b* = 70.94, *t*(69) = 2.90, 95% CI [22.14, 119.73], *p* = .005, and with GI, *b* = 243.05, *t*(69) = 2.93, 95% CI [77.84, 408.25], *p* = .005, but not with QI, *b* = 67.55, *t*(69) = 1.65, 95% CI [-14.01, 149.11], *p* = .103. Lastly, similar to the manually rated joint action, there was no effect of movement synchrony on the PANAS, neither the positive items, *b* = -32.21, *t*(69) = -0.80, 95% CI [-112.69, 48.27], *p* = .427, nor for the negative items, *b* = -18.22, *t*(69) = -1.27, 95% CI [-46.91, 10.46], *p* = .209.

## 4 Discussion

Socially anxious individuals have consistently been shown to be less liked by others (e.g., Meleshko & Alden, 1993; Pilkonis, 1977). In the present study, it was hypothesised that engagement in joint action is likely to be reduced in high socially anxious individuals compared to low socially anxious individuals and that this might partially account for less positive reactions from other people. We applied two methods for analysing joint action and our results are two-sided. The conventional way of analysing joint action (Bernieri, 1988) provide support for this hypothesis. In conversations with another person, high socially anxious individuals elicited less positive responses from their conversation partner and they showed reduced joint action. Crucially, mediation analysis showed that the differences in responses elicited by high and low socially anxious individuals can be accounted for by differences in joint action. In particular, joint action partially mediated the effect of social anxiety on the conversation partner's overall ratings of the interaction, the extent to which they liked the person they were talking to, and their desire to meet them again. From this perspective, it therefore appears that less engagement in complementary behaviour meshing with one's partner's actions helps explain why socially anxious individuals are perceived less positively by other people.

On the contrary, findings from the Motion Energy Analysis suggest otherwise. Movement synchrony defined as differences in consecutive video-frames in the ROIs (i.e., motion energy) was found not to be associated with level of social anxiety. It was positively related, however, to desire for future interaction and general impression of the person the CP just talked to. In other words, the more movement synchrony the person engaged in, the more positively did the CP rate the HSA or LSA individual. This lends further support to previous research that found that movement synchrony appears to be essential for rapport and good relationship quality in various contexts (e.g. Bernieri, 1988; Bernieri et al., 1988; Ramseyer & Tschacher, 2011; Tunçgenç & Cohen, 2016). Yet, with this automated and rather new technique, our hypothesis that high social anxiety is linked to reduced movement synchrony could not be supported.

In both analyses conversation partner did not experience an effect on their affectivity after talking to LSA or HSA individuals. The PANAS was the only measure used that did not ask about the conversation or the (non-) anxious individual directly but exclusively about how the CP her- or himself felt. Possibly, it takes more to significantly change the CP's mood and feelings than a short 5-min conversation with someone they just met. The same applies to movement synchrony and joint action not being associated with the PANAS.

The opposing results of an association between social anxiety and joint action or movement synchrony raise questions about the optional ways of measuring joint action. The MEA software belongs to “frame-differencing methods”, which have been argued to be the most promising non-verbal synchrony techniques (Paxton & Dale, 2013), as it is observer-independent once the procedure is set (Ramseyer & Tschacher, 2011) and allows objective quantitative measures of dynamic movement coordination through the empirical analysis of digitalised film material such as simultaneous movement and tempo similarity. However, we argue that it does not allow the assessment of meaningful Gestalt-patterns like Bernieri's ratings (1988) do, which interestingly also showed the highest interrater reliability. Movement synchrony as measured by the MEA may thus only measure one part of the concept of joint action. The Gestalt approach allows to take the holistic, dance-like coordination and collaborative smoothness (e.g., alignment of posture to complement the conversation partner; Bekkering et al., 2009; Garrod & Pickering, 2009) of the interaction into account rather than correlating individual movements. Moreover, these concepts include the meaning of movements and thus, prior knowledge of meaning of movement and behaviour. Although an automated software package like MEA might be useful in disentangling two of the components, it will not be able to pick up on the crucial element of joint action, namely, gestalt-like smoothness. In our view, extended validation studies are required in future to investigate to what extent these new automated techniques such as the MEA (Ramseyer & Tschacher, 2011) can be used to measure social concepts like joint action. The present study focused on joint action in non-verbal behaviour. As Mein, Fay, and Page (2016) have demonstrated that individuals with social anxiety disorder also show reduced verbal joint action, it seems likely that such reductions in both verbal and non-verbal joint action, beyond movement synchrony as defined by motion energy, are likely to contribute to the less positive reactions that socially anxious individuals elicit from other people.

Bernieri (1988) argues that successful joint action depends on individuals being able to consistently focus their attention on the person they are talking with so that they can coordinate with, and complement, the other person's actions in a precisely timed manner. Consistent with this view, Mein et al. (2016) found that asking low socially anxious individuals to perform a dual attention task led to a reduction in joint action.

A considerable body of research (see Clark, 2005; Clark & Wells, 1995; Hofmann, 2007; Rapee & Heimberg, 1997) shows that individuals with SAD adopt an egocentric processing mode. They are often self-focused and engage in self-protective strategies (i.e., safety behaviours) that are likely to reduce the mental resources that they have available for focusing on another person and for engaging in joint action. It therefore seems likely that an egocentric processing mode is part of the explanation for socially anxious individuals having difficulty with joint action. A formal test of this explanation would require studies that manipulate safety behaviours and/or self-focus and investigate the effect of such manipulations on joint action. We are not aware of any such studies. However, several studies have shown that certain types of safety behaviour contribute to the less positive reactions that socially anxious individuals elicit from others. Collins and Miller (1994) found that self-disclosure is related to the development of likeability, something that high socially anxious individuals appear less likely to engage in (Voncken & Dijk, 2013). Similarly, both Hirsch, Meynen and Clark (2004) found that socially anxious individuals who used more avoidant safety behaviours (such as “say little”) in a conversation were rated more negatively by their conversation partner. Interestingly, this effect was not found for impression management safety behaviours (such as “checking that you are coming across well”), perhaps because these safety behaviours require the socially anxious individual to pay some attention to the other person.

After many inconsistent findings in studies of specific behavioural differences between high and low anxious participants regarding intrapersonal processes (e.g., frequency and duration of speech and gesture, and fidgeting; Pilkonis, 1977; Segrin & Kinney, 1995; Bakerman & Edel, 2002; Heerey & Kring, 2007), the focus on interpersonal processes in this study sheds light on what makes anxious individuals less liked. The present study adds well to the findings of Mein et al. (2016) and Varlet et al. (2014), as it investigated joint action as a non-linguistic process, exclusively, in unstructured naturalistic interactions, whereas Varlet used hand held pendulums to investigate movement coordination in individuals with social anxiety disorder. Studying naturalistic unstructured conversations seems to be more relevant than structured interactions such as speeches to understand social anxiety (e.g., Rapee & Lim, 1992; Voncken & Bögels, 2008). Additionally, it may be mainly unstructured conversations and interactions out of which social relationships possibly develop as Mein (2013) suggests. Future research should also investigate mechanisms of reductions in joint action on different levels, including neurological processes (e.g., mirror neurons; Dumas, Nadel, Soussignan, Martinerie, & Garnero, 2010).

*Limitations:* Some potentially relevant methodological issues were not fully addressed. First, it could be argued that LSA participants and CP may have had more similar facial expressions during the conversation and this may have influenced the judges’ ratings of joint action. As we did not assess facial similarity, we were not able to directly assess this suggestion. However, it seems unlikely that this confound would have had a major influence, even if it was present. Bernieri et al. (1994) found that rated synchrony patterns were virtually the same between judges who rated movement synchrony while watching the normal video display without sound (i.e., as was done in the present study) and judges who rated movement synchrony while watching mosaic video displays where facial affect and other fine details were removed beforehand.

Second, it may be a limitation that only two judges rated joint action manually. Nevertheless, the interrater reliability in the present study was comparable to the ones attained in previous studies (e.g., Bernieri, 1988; Bernieri et al., 1988; Mein, 2013).

Third, as the study used an analogue sample, it is unclear whether the results will generalise to a clinical population of social anxiety. Yet, existing research supports the use of analogue samples from the general population as a valid strategy for identifying processes relevant in social anxiety disorder as non-clinically high socially anxious individuals often have similar patterns of physiological and cognitive processes during social interactions (Stopa & Clark, 2001). Moreover, there was substantial overlap between the CP and LSA groups based on the SIAS cut-off scores. We cannot therefore exclude that CPs may have rated LSA participants more positively because of the similarity in this regard (same level of social anxiety; Byrne & Griffitt, 1966; Collins & Miller, 1994). However, on the BFNE-II the conversation partner significantly scored higher than the LSA but lower than HSA group, which is what we intended with the previously specified cut-off scores on the SIAS screening tool. Nevertheless, future research should repeat the present study with varied scores on the SIAS.

Finally, future research should investigate a direct link between egocentric focus (self-focused attention and monitoring, and the use of safety behaviours) and poorer joint action as egocentric focus has not been directly addressed in the present study.

*Conclusion*: The goal of the present study was to shed more light on interpersonal processes that contribute to the reduced likeability of individuals with social anxiety. Poor engagement in joint action mediated the relationship between social anxiety and desire for future interaction, quality of interaction, as well as general impression. These results are in line with cognitive behavioural models of social anxiety emphasising the increased self-focused attention, which leaves anxious individuals with fewer cognitive resources to commit oneself to-the ongoing conversation and thereby, increasing the likelihood of evoking negative reactions from others (Clark, 2005; Rapee & Heimberg, 1997). Consequently, the vicious cycle present in social anxiety disorder is perpetuated. Cognitive behavioural therapy interventions, such as the task concentration training, may be used to help to change such self-focus into (social) task-focus (e.g., a conversation) for those with social anxiety disorder (Bögels, 2006; Mulkens, Bögels, de Jong, & Louwers, 2001).

The present findings are especially promising as they might pave the way of future research focusing more on interpersonal processes and taking a holistic Gestalt approach. In order to fully understand and more effectively treat social anxiety, the involvement of person, interlocutor and situation must be taken into account, contrary to traditionally taking an independent and intrapersonal approach (Kashdan & Savostyanova, 2011).

## Declaration of interest

Declarations of interest: none.

## Funding

This research was supported by a grant Wellcome Trust to A. Ehlers & D.M. Clark (069777). The funding source had no involvement in study design; the collection, analysis and interpretation of data; in the writing of the report; or in the decision to submit the article for publication.

## Footnotes

3 When combining the three measures, similar results were observed.
