# Supplementary material for: Disrupted joint action accounts for reduced likability of socially anxious individuals
Source: J Behav Ther Exp Psychiatry. 2020 Sep;68:101512. doi: 10.1016/j.jbtep.2019.101512 (PMC7232015; doi:10.1016/j.jbtep.2019.101512)
Supplement: Multimedia component 1 [file mmc1.docx]

**Supplementary Material**

Traditionally, the analysis of nonverbal synchrony required researchers to manually code single frames of sound-film recordings (e.g., Condon & Ogston, 1966) or to train judges to do holistic ratings (e.g., Bernieri, 1988), which is what we did for the first part. Nowadays, technical advances also enable objective measurement that is cost-effective and efficient (Paxton & Dale, 2013). Motion energy analysis (MEA; Ramseyer & Tschacher, 2011; Version 4.03a) was used as a second method to quantify movement synchrony (i.e., joint action). Prerequisites for the recordings are stable light conditions, a static camera position and digitized video recording of each conversation. The software allows to automatically and continuously monitor any changes of movement by detecting frame-by-frame changes in previously specified regions of interest (ROI) in the video footage of each conversation. Thus, motion energy is defined as difference between consecutive video-frames in transformed gray scale pixels in the ROIs (Grammer, Honda, Juette, & Schmitt, 1999; Tschacher, Rees, & Ramseyer, 2014). Everything else has to remain static so that every pixel change from frame to frame can be traced back to interlocutors’ movement (Paxton & Dale, 2013). In the present study, two ROIs were defined: the CP’s and their partner’s (i.e., HSA or LSA) whole body, covering head and legs (see Figure 2), similar to the manual behavioural analyses. Movement was detected at a resolution of 25 frames per second. Each conversation lasted 5 minutes. Time series of frame-by-frame movement quantity in the ROIs (i.e., motion energy) were cross-correlated for time windows of 30 seconds that did not overlap to account for short turn-taking latencies during a small talk interaction (Nelson, Grahe, Ramseyer & Serier, 2014; Tschacher et al., 2014). The cross-correlations were time-lagged ±5 for each window. That is, the correlation coefficient is computed for each time lag relative to the other between the two interlocutors’ time series. The correlation will be highest closer to a lag of 0 if the movements of the two individuals are synchronised in time (for a detailed description of cross-correlation, see Paxton & Dale, 2013). Cross-correlations were then standardised with Fisher’s Z and their absolute values were aggregated over the 5-minute interval. At a time window of plus and minus 5 seconds and 25 frames per second, this results in 251 correlations per minute and a total of 1255 correlations for five minutes. The use of absolute values implies that both positive and negative cross-correlations contribute to represent movement synchrony. By averaging all correlation coefficients, a global movement coordination score between CP and HSA or LSA could be obtained (Galbusera, Finn, & Fuchs, 2018; Nelson et al., 2014). This score was used as a second automated measure of movement synchrony and employed for further analyses.


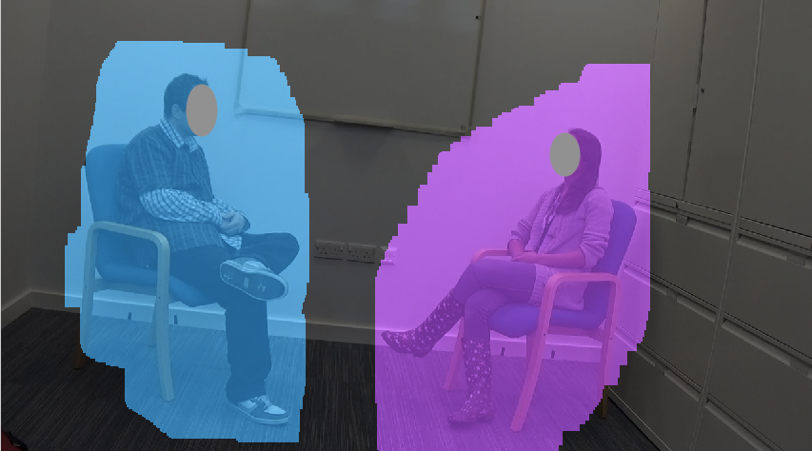


*Figure 1.* Motion Energy Analysis of Predefined Regions of Interest (ROIs)


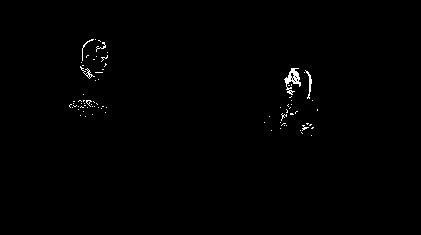


*Figure 2.* Gray-pixel frames in Predefined Regions of Interest (ROIs)


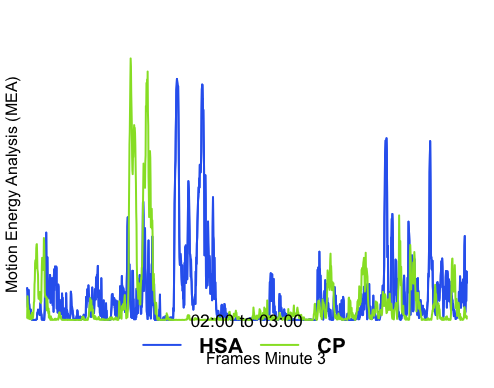


*Figure 3.* Motion Energy Analysis during Minute 3 of HSA Participant 1 and CP.

References

Bernieri, F. J. (1988). Coordinated movement and rapport in teacher-student

interactions.*Journal of Nonverbal Behavior*,*12*(2), 120-138. doi:10.1007/BF00986930

Condon, W. S., & Ogston, W. D. (1966). Sound film analysis of normal and pathological

behavior patterns. *Journal of nervous and mental disease*.

Galbusera, L., Finn, M. T., & Fuchs, T. (2018). Interactional synchrony and negative symptoms: An outcome study of body-oriented psychotherapy for schizophrenia. *Psychotherapy Research*, *28*(3), 457-469. doi:10.1080/10503307.2016.1216624

Grammer, K., Honda, M., Juette, A., & Schmitt, A. (1999). Fuzziness of nonverbal courtship communication unblurred by motion energy detection. *Journal of personality and social psychology*, *77*(3), 487. doi:10.1037/0022-3514.77.3.487

Nelson, A., Grahe, J. E., Serier, K., & Ramseyer, F. (2014). Psychological data from an exploration of the rapport/synchrony interplay using motion energy analysis. *Journal of Open Psychology Data*, *2*(1), e5. doi:10.5334/jopd.ae

Paxton, A., & Dale, R. (2013). Frame-differencing methods for measuring bodily synchrony in conversation. *Behavior research methods*, *45*(2), 329-343. doi:10.3758/s13428-012-0249-2

Ramseyer, F., & Tschacher, W. (2011). Nonverbal synchrony in psychotherapy: Coordinated

body movement reflects relationship quality and outcome. Journal of Consulting and Clinical Psychology, 79(3), 284-295. doi:10.1037/a0023419

Tschacher, W., Rees, G. M., & Ramseyer, F. (2014). Nonverbal synchrony and affect in dyadic interactions. *Frontiers in psychology*, *5*, 1323. doi:10.3389/fpsyg.2014.01323
